# Cellular dosimetry of [^177^Lu]Lu-DOTA-[Tyr^3^]octreotate radionuclide therapy: the impact of modeling assumptions on the correlation with in vitro cytotoxicity

**DOI:** 10.1186/s40658-020-0276-5

**Published:** 2020-02-10

**Authors:** Giulia Tamborino, Marijke De Saint-Hubert, Lara Struelens, Dayana C. Seoane, Eline A. M. Ruigrok, An Aerts, Wiggert A. van Cappellen, Marion de Jong, Mark W. Konijnenberg, Julie Nonnekens

**Affiliations:** 1 Research in Dosimetric Application, Belgian Nuclear Research Centre (SCK•CEN), Mol, Belgium; 2 Department of Radiology & Nuclear Medicine, Erasmus MC, Rotterdam, The Netherlands; 3 Department of Experimental Urology, Erasmus MC, Rotterdam, The Netherlands; 4 Radiobiology Unit, Belgian Nuclear Research Centre (SCK•CEN), Mol, Belgium; 5 Erasmus Optical Imaging Centre, Erasmus MC, Rotterdam, The Netherlands; 6 Department of Molecular Genetics, Erasmus MC, Rotterdam, The Netherlands; 7 Oncode Institute, Erasmus MC, Rotterdam, The Netherlands

**Keywords:** Cellular dosimetry, [^177^Lu]Lu-DOTA-[Tyr^3^]octreotate, Polygonal mesh, *S* values, in vitro cytotoxicity correlation

## Abstract

**Background:**

Survival and linear-quadratic model fitting parameters implemented in treatment planning for targeted radionuclide therapy depend on accurate cellular dosimetry. Therefore, we have built a refined cellular dosimetry model for [^177^Lu]Lu-DOTA-[Tyr^3^]octreotate (^177^Lu-DOTATATE) in vitro experiments, accounting for specific cell morphologies and sub-cellular radioactivity distributions.

**Methods:**

Time activity curves were measured and modeled for medium, membrane-bound, and internalized activity fractions over 6 days. Clonogenic survival assays were performed at various added activities (0.1–2.5 MBq/ml). 3D microscopy images (stained for cytoplasm, nucleus, and Golgi) were used as reference for developing polygonal meshes (PM) in 3DsMax to accurately render the cellular and organelle geometry. Absorbed doses to the nucleus per decay (*S* values) were calculated for 3 cellular morphologies: spheres (MIRDcell), truncated cone-shaped constructive solid geometry (CSG within MCNP6.1), and realistic PM models, using Geant4-10.03. The geometrical set-up of the clonogenic survival assays was modeled, including dynamic changes in proliferation, proximity variations, and cell death. The absorbed dose to the nucleus by the radioactive source cell (self-dose) and surrounding source cells (cross-dose) was calculated applying the MIRD formalism. Finally, the correlation between absorbed dose and survival fraction was fitted using a linear dose-response curve (high *α*/*β* or fast sub-lethal damage repair half-life) for different assumptions, related to cellular shape and localization of the internalized fraction of activity.

**Results:**

The cross-dose, depending on cell proximity and colony formation, is a minor (15%) contributor to the total absorbed dose. Cellular volume (inverse exponential trend), shape modeling (up to 65%), and internalized source localization (up to + 149% comparing cytoplasm to Golgi) significantly influence the self-dose to nucleus. The absorbed dose delivered to the nucleus during a clonogenic survival assay is 3-fold higher with MIRDcell compared to the polygonal mesh structures. Our cellular dosimetry model indicates that ^177^Lu-DOTATATE treatment might be more effective than suggested by average spherical cell dosimetry, predicting a lower absorbed dose for the same cellular survival. Dose-rate effects and heterogeneous dose delivery might account for differences in dose-response compared to x-ray irradiation.

**Conclusion:**

Our results demonstrate that modeling of cellular and organelle geometry is crucial to perform accurate in vitro dosimetry.

## Background

Targeted radionuclide therapy (TRT) is a promising treatment for solid tumors and micro metastases [1]. Patients with metastasized neuroendocrine tumors (NETs) overexpressing the somatostatin receptor type 2 (SST_2_) can be treated with peptide receptor radionuclide therapy (PRRT). PRRT with the radiolabeled somatostatin receptor agonist DOTA-[Tyr^3^]octreotate ([^177^Lu]Lu-DOTA-[Tyr^3^]octreotate or ^177^Lu-DOTATATE) has successfully been employed in the past years [2].

Clinical optimization of TRT most often relies on the evaluation of the absorbed dose-effect relationship in pre-clinical settings aiming to assess efficacy and toxicity of the treatment. The fundamental knowledge derived from a better understanding of the action of ionizing radiation on biological matter through the development of cellular dosimetry may provide novel and more effective strategies for TRT treatment delivery. However, biological effects from in vitro experiments are mainly reported in direct correlation with the added activities (in MBq/ml), hindering the prediction and comparison of therapeutic efficacy of different radiopharmaceuticals.

For this purpose, the Medical Internal Radiation Dose (MIRD) committee has developed a general formalism to convert administered activities into absorbed doses based on the *S* value [3], i.e., the absorbed dose-rate to a target region per unit activity from a source region. This concept, initially adopted at organ level, has been extended to the cellular level leading to the creation of a database of cellular *S* values for several cell/nucleus radii and radionuclides incorporated into different compartments [4] included in MIRDcell [5], an applet software application.

This tool, however, has several limitations related mainly to simplified biological assumptions (e.g., spherical cell geometry lacking a physical membrane, unit density, and uniform activity distributions) and a semi-analytical radiation transport model adopting the continuous-slowing-down approximation (CSDA), thus neglecting electron straggling and secondary electrons. Indeed, other authors made use of pre-calculated Dose Point Kernels [6, 7] or direct Monte Carlo radiation transport [8–10], pointing out the discrepancy with MIRDcell, specifically for the low energy range of electrons [11]. Moreover, it was demonstrated that asymmetries in the geometry [12, 13], as well as non-concentric cell and nucleus morphology [14] significantly impact the absorbed dose to the nucleus. Hence, a realistic geometrical representation of the cell, including organelles that can play a key role in the re-localization of the radiopharmaceutical product, such as the Golgi apparatus, is indispensable to perform proper cellular dosimetry. The Golgi apparatus is an intracellular membrane system located near the cell nucleus and responsible for processing of proteins. After receptor agonist stimulation, the SST_2_ will be internalized into the cell and is either directly recycled to the plasma membrane or is relocated to the trans-Golgi network [15]. It remains yet to be determined whether the receptor agonist remains bound to the receptor during the relocation or if it is released; however, the impact of the radiopharmaceutical localization in the Golgi, besides cytoplasm and membrane, has never been assessed.

Further development is needed in cellular dosimetry to investigate reliable dose-effect relationships for cell survival. These models can then be integrated into treatment planning systems for TRT. Conversely, in external beam radiotherapy (EBRT) the well-established linear-quadratic (LQ) model [16] is used to describe the response to radiation; however, it remains yet to be determined if the LQ-model would be able to describe the variable low-dose rate and heterogeneous dose delivery characterizing PRRT.

Hence, the present work aims to build a more refined dosimetry model, based on the MIRD formalism, for in vitro cell experiments with ^177^Lu-DOTATATE and correlate the cellular absorbed doses to cell survival in order to compare it to x-ray exposure and LQ-model prediction.

## Methods

### Cell lines and treatment

Experiments were performed with human osteosarcoma cells (U2OS) stably expressing somatostatin receptor type 2 (U2OS+SST_2_) and maintained as previously described [17]. For uptake and survival experiments, cells were treated with different activity quantities of ^177^Lu-DOTATATE (IDB Holland). Molar activity was 53 MBq/nmol, radiometal incorporation > 95% and radiochemical purity > 90%.

### Immunofluorescent staining and imaging

Cells were grown on quartz coverslips (Xantec bioanalytics GmbH, Düsseldorf, Germany) in 6-well plates until ~ 25% confluency and fixed with 2% paraformaldehyde (Sigma Aldrich) for 15 min at room temperature (RT), permeabilized for 20 min at RT in PBS containing 0.1% Triton X-100 (Sigma Aldrich), and incubated in blocking buffer (PBS, 0.1% Triton X-100, 2% bovine serum albumin (Sigma Aldrich)) for 30 min at RT. Next, cells were incubated for 90 min at RT with the primary antibody, rabbit anti-Giantin (PRB-114C BioLegend, San Diego, CA, USA, 1/1000) diluted in blocking buffer. Following incubation, cells were washed with PBS 0.1% Triton X-100 and incubated with 100nM SiR-actin (SC001 Spirochrome) and the secondary antibody (goat anti-rabbit Alexa Fluor 488 1/1000) in blocking buffer for 90 min at RT. Cells were washed with PBS and incubated with 1 μg/ml propidium iodide (Sigma Aldrich) and 10 μg/ml RNase in PBS for 30 min at RT. Cells were washed with PBS and mounted in 87% glycerol pH8.6 (Sigma Aldrich). *Z*-stack imaging was performed using a 4Pi confocal microscope (Leica, Mannheim, Germany) and images were analyzed using the ImageJ software [18].

### Uptake assay

U2OS+SST_2_ cells were seeded in 12-well plates and the next day cells were incubated with 0.5, 1, and 2.5 MBq/ml of 177Lu-DOTATATE in 1 mL medium for 15 min up to 4 h at 37 °C, 5% CO_2_. Subsequently, cells were washed with PBS. For short-term measurements, samples were collected every 15 min. The membrane-bound fraction was collected by incubating cells for 10 min in 1 mL 50 mM glycine (Sigma Aldrich) and 100 mM NaCl (Sigma Aldrich), pH 2.8. The internalized fraction was collected by lysing the cells 1 mL 0.1 M NaOH (Sigma Aldrich). For day 1–6 measurements, a new medium was added to the cells and they were incubated at 37 °C, 5% CO_2_. For every time point, medium, membrane-bound, and internalized fractions were collected. This data was combined with the uptake data (2.5 MBq/ml) from the previous study [17]. Gamma counter measurements were corrected for decay and the uncertainty on estimated activity fractions in the different cell compartments was calculated as one standard deviation of 2 independent experiments, each performed in triplicate. Fractions of added activity were also determined for activities of 0.1 and 0.25 MBq/ml by 3D inverse distance weighting extrapolation using Python [19]. Furthermore, total cell number per well was measured using a CASY cell counter (OMNI Life Science).

### Clonogenic survival assay

Cells were incubated with 0.1-2.5 MBq/ml of ^177^Lu-DOTATATE for 4 h and clonogenic potential was measured at 8 days after treatment as previously described [17]. New data was combined with survival data from the previous study by Nonnekens et al. [17] to investigate the reproducibility of these experiments.

In order to compare these results to external irradiation, cells were seeded in a 6-well plate and 6 h later irradiated with 86 keV x-ray (0.5–4 Gy). Clonogenic potential was measured at 8 days after treatment.

### Monte Carlo simulations: cellular shape assumptions and software choice

**Fig. 1:**
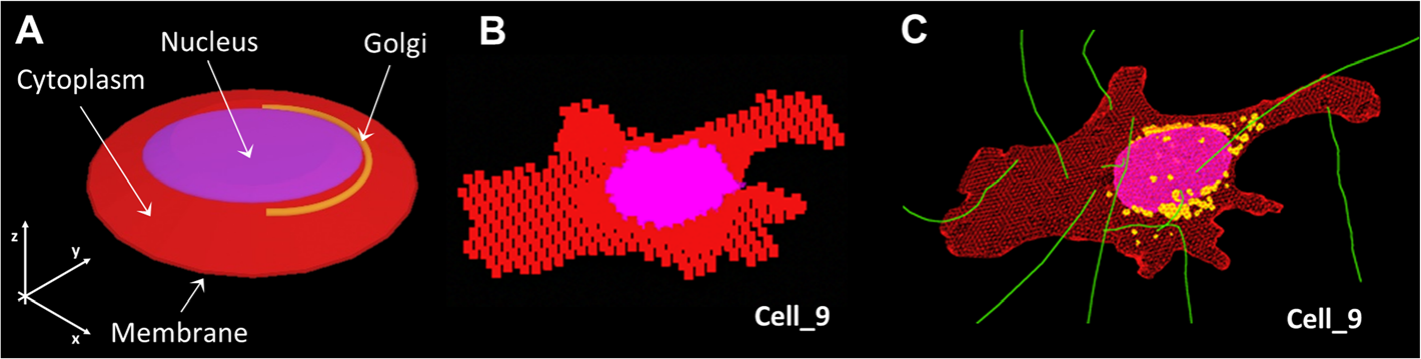
Cellular shape modeling assumptions: **a** Truncated cone, **b** Voxelized geometry, **c** Polygonal mesh structure

Three geometrical modeling assumptions were used to render the cellular shape, as obtained through 4Pi confocal microscopy imaging: MIRDcell (spheres), truncated cone-shaped constructive solid geometry (CSG) and realistic cell representations, based on (1) voxels (2.77 μm × 2.77 μm × 1.3 μm) and (2) polygonal mesh structures (PM) (2D interconnected surfaces) (Fig. 1). The CSG-based shape was constructed as reported in Table 1 and will be referred to as truncated cone geometry.

**Table 1:**
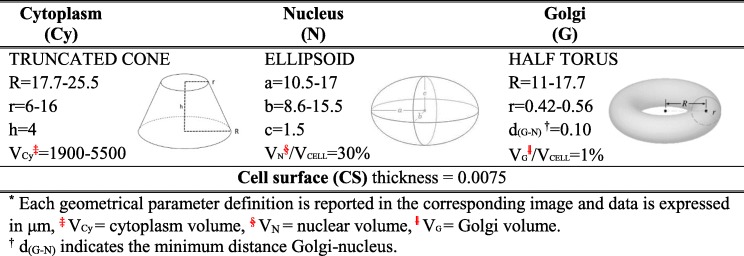
Shapes and range of dimensions characterizing the CSG-shaped cell

*Each geometrical parameter definition is reported in the corresponding image and data is expressed in μm, ^‡^ *V*Cy = cytoplasm volume, ^§^ *V*_N_ = nuclear volume, ǁ *V*G = Golgi volume. ^†^ *d*_(G-N)_ indicates the minimum distance Golgi-nucleus

In order to calculate the *S* values for the truncated cone and voxelized geometries, the F6 tally (i.e., calculation of the absorbed dose in kerma approximation) was used within the Monte Carlo N-Particle 6.1 code [20]. The minimum voxel-size was limited by internal source sampling. The electron cut-off energy was lowered to 100 eV and 20 eV to extend the electron transport to microscopic scales [21].

**Fig. 2:**
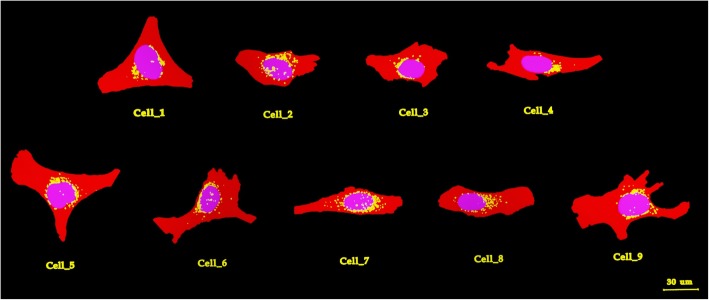
Polygonal Mesh (PM) models in 3Ds Max. Cytoplasm, Golgi and nucleus are shown in red, yellow and blue, respectively. The scale bar is 30 μm

Currently, MCNP 6.1 does not allow to import PMs, therefore Geant4 10.3 [22] was used instead. 3Ds Max [23] was used to model the specific morphology of cytoplasm, nucleus and Golgi of 9 4Pi confocal microscopy 3D images (Fig. 2), while the shell operator was employed to add the cell surface. Each cellular structure was saved with the highest numerical precision available (12 decimals) and converted into GDML format with FASTRAD [24]. “Penelope” low energy physics model [25] was adopted in Geant4 to track particles down to an energy of 250 eV (cut-off range of 10 nm) while the default production threshold of secondary electrons was lowered to 1 nm and the step size was limited to 1 μm when needed.

The chemical composition of the cell nucleus was taken to be that of normal cells [26], while cytoplasm and Golgi were considered as water (*ρ* = 1 g/cm^3^) and cell membrane as lipid (*ρ* = 0.92 g/cm^3^). The *β*-spectrum of lutetium-177 was sampled according to RADAR decay data (starting at 3.8 keV) since only electrons above 6 keV are expected to travel at least a distance of 1 μm (average Golgi-nucleus path) [11]. Auger- and internal conversion electron data were taken from ICRP107 [27]. The radionuclide was considered to be uniformly distributed in cell growth medium (M), cell membrane, cytoplasm or Golgi. The number of particles run per simulation ensured a relative error below 1%. A computational cluster providing 648 CPU cores (MCNP6.1) and a relatively high-end hardware (Intel Xeon W-2133 CPU, NVIDIA Quadro P4000 CPU, 32 GB RAM) for Geant4-10.3 and 3Ds Max were used.

### Parameter analysis of *S* values

**Fig. 3:**
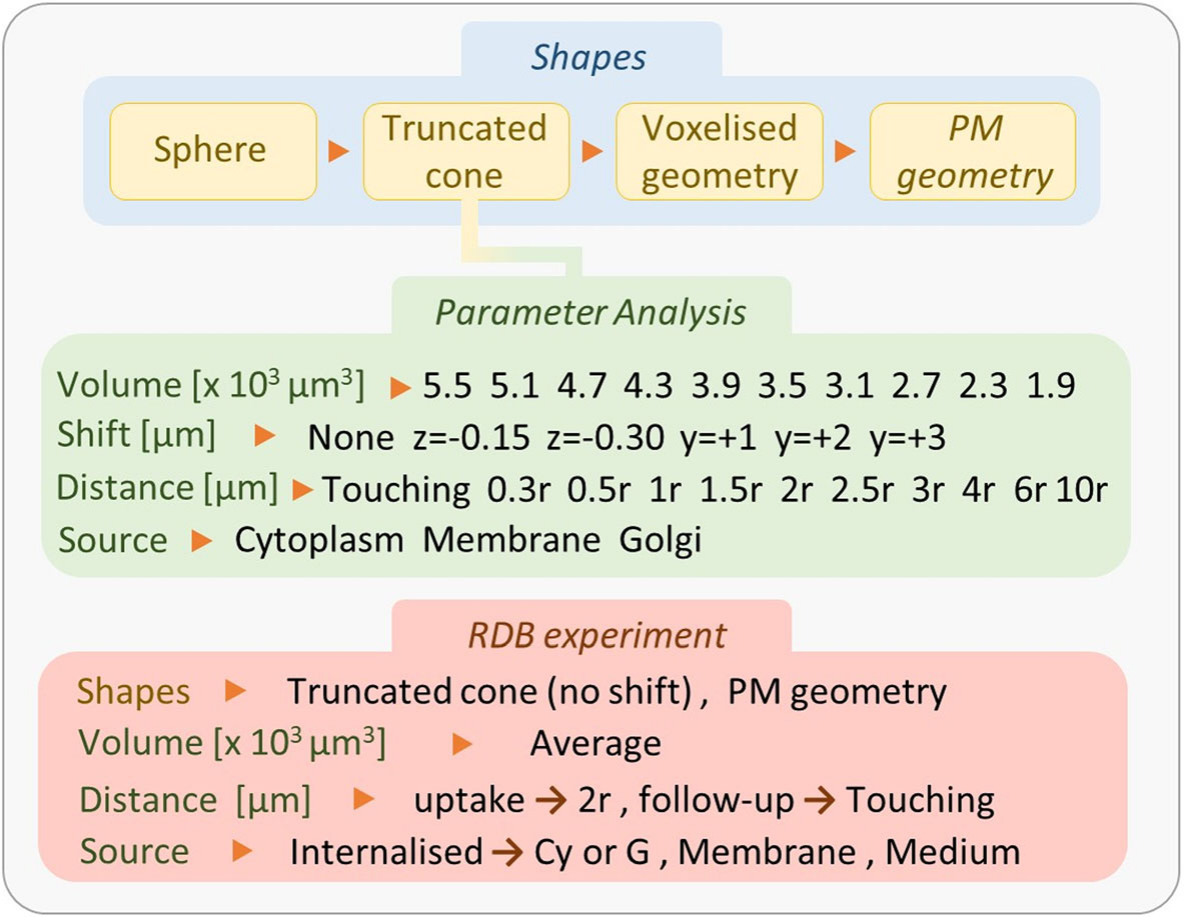
Schematic summary of the simulations performed for the parameter analysis with the truncated cone geometry (green area) and the in vitro experiment (red area). The position shifts of the nucleus are referred to the *z*- and *y*-axis. The relative distance between cells is indicated as multiples of the cell radius (“r”). Regarding the simulation of the in vitro experiment, during the 4-h uptake, the distance between cells was assumed constant and evaluated on average (1 cell diameter); in the next 6 days, cells were able to form colonies made of neighboring cells

The truncated cone geometry was generated in MCNP6.1 to assess the influence of several cell dimensions (1900–5500 um^3^, as observed from the cell sample), nucleus position within the cell (on 2 coordinates), cell-to-cell proximity (from 0 to 5 cell diameters), and radiation source location (cytoplasm, cell membrane, Golgi) on the calculated *S* values considering the nucleus as target volume (Fig. 3). Cells were placed adherent to the bottom of one of the wells in a 12-well culture dish plate and adjacent to each other (excluding cell proximity analysis where they are equally spaced). In all simulations, the total number of cells was fixed on the basis of the average range of beta particles emitted by lutetium-177 (280 μm). The total cross-dose was scored on the nucleus of the central cell, placing the activity in the selected compartment of all the surrounding ones.

### Absorbed dose calculations

The absorbed dose to the nucleus (D(N)) for all the cellular geometries was calculated applying the MIRD formalism:

1 $$ D(N)={\widetilde{\mathrm{A}}}_M\times {S}_{N\leftarrow M}+{\widetilde{\mathrm{A}}}_{CS}\times {S}_{N\leftarrow CS}+{\widetilde{\mathrm{A}}}_C\times {S}_{N\leftarrow C} $$

where *S*_*N* ⟵ *M*_, *S*_*N* ⟵ *CS*_ and *S*_*N* ⟵ *C*_ are the absorbed doses to the nucleus per decay from medium, cell membrane, and inside the cell (cytoplasm or Golgi). The cumulated activities inside the cell $\left({\widetilde{A}}_C\right)$, membrane bound $\left({\widetilde{\mathrm{A}}}_{CS}\right)$ and in medium $\left({\widetilde{A}}_M\right)$ were evaluated in the first 4 h integrating the time-activity curves; in the next 6 days, they were evaluated as follows:

2 $$ {\widetilde{\mathrm{A}}}_{M,C\  or\  CS}={f}_{M,C\  or\  CS}\times {\int}_{T_1}^{T_2}{A}_0\times {e}^{-{\lambda}_pt} dt $$

where *A*_0_ is the initial added activity, *T*_1_ and *T*_2_ correspond to time intervals of 24 h, except for the first day lasting 20 h, and λ_p_ is the physical decay constant of the radiopharmaceutical; *f*_*M*,_ *f*_*C*,_ *f*_*CS*_ are the fractions of activity localized in medium, cell, or membrane, respectively, and corrected for decay and cell growth ( *f*_*C*_ and *f*_*CS*_ divide the cumulated activity per number of cells measured in each day). The absorbed dose to the radioactive cell (self-dose) and to each one of the neighboring cells (cross-dose) was determined; the total cross-dose was reported.

### Simulation set-up of the clonogenic survival assay

**Fig. 4:**
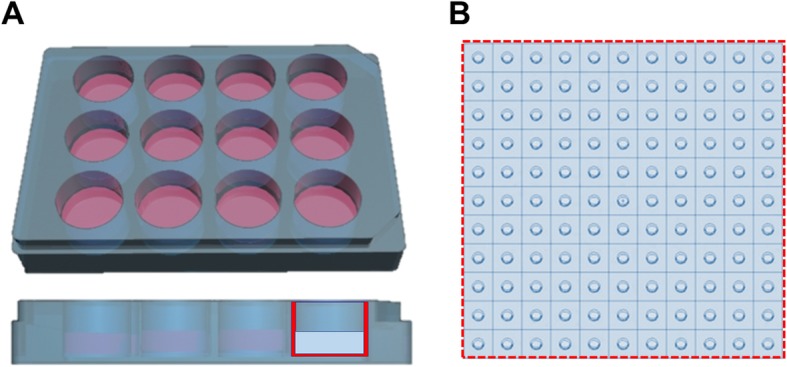
Representation of the simulation environment. **a** Rendering of a 12 well plate with the simulated cylindrical geometry superimposed on the side view. **b** Section of the bottom of the cylindrical well with the reference cell sample (4 h set-up) lying on top

During the 4 h uptake the distance between cells was assumed constant and evaluated on average (1 cell diameter) (Fig. 4). Both self- and cross-dose from equally spaced cells was evaluated, assuming an infinite medium. In the next 6 days, the distance between cells was changed according to the proliferation rate going from isolated cells to clusters of cells of increasing sizes, depending on the day. Only the progeny was considered to contribute to the cross-dose. All cells were assumed to be clonogenic with a doubling time of 27–44 h calculated using the growth rate obtained by SRB assay [17]. The activity was assumed to be homogenously distributed among the cell population and equally split to the offspring. The fraction of activity localized inside the cell $\left(\ {f}_C,{\widetilde{A}}_C\right)$ was assumed to be distributed either in the cytoplasm $\left({f}_{CY},{\widetilde{A}}_{CY}\right)$ or in the Golgi $\left(\ {f}_G,{\widetilde{A}}_G\right)$.

### Statistical methods

The absolute error on the absorbed dose was evaluated by propagating the error obtained as one standard deviation (SD) of the following components:

- Fractions of activities (5–33%)
- Cell counts (11–18%)
- Added activities (1–3%)
- Variance in *S* value due to 9 different cell shapes (18–27%)

Selection of the most likely curve fitting result was performed using the Akaike Information Criterion (AIC) that takes the goodness of fit and degrees of freedom into account; fitting was performed according to the least square method, with Pearson’s *R*^2^ as parameter for its goodness (*R*^2^ > 0.7).

The paired *t* test was used to assess the significant difference (*p* < 0.05) between sets of data within the shape modeling comparison and parameter analysis.

## Results

### Parameter analysis

#### Simplified cellular shapes (MIRDcell) result in higher self *S* values than PM geometry

In previous studies, simplified geometries reproducing cellular morphologies were compared to analyze the impact of shape-modeling on the *S* values; therefore we have further explored this comparison including, as reference, the reconstruction of 9 confocal microscope images of U2OS+SST_2_ cells with voxels and polygonal meshes.

The *S* values comparison between different cellular morphologies (MIRDcell, truncated cone, voxel structures, and PM models) with comparable volumes is reported in Additional file 1: Table S1. In summary, the truncated cone geometry reduces the *S* values for N ← Cy and N ← CS on average by 39 ± 3% (*p* < 0.05) and 13 ± 10% (*p* < 0.05), respectively, compared to spheres. PM geometries decrease these differences further to 60 ± 6% (*p* < 0.05) for N ← Cy and 37 ± 5% (*p* < 0.05) for N ← CS, compared to spheres. As expected, there is no significant difference in the *S* value when comparing the most realistic cell representations: voxelized and PM structures (*p* > 0.05); specifically, the average relative percentage difference (RPD) between the two sets of *S* values is 14.8%. The *S* values for the PM models are reported in Table 2.

**Table 2:** Self-dose from cytoplasm, Golgi, and cell membrane to nucleus for each of the 9 PM geometries

Cell *n*^° a^	V_C_^c^ [*μm*^3^]	V_N_^d^ [*μm*^3^]	S_(N ← Cy)_ $\left[\frac{Gy}{Bq\ s}\right]$	S_(N ← G)_ $\left[\frac{Gy}{Bq\ s}\right]$	S_(N ← CS)_ $\left[\frac{Gy}{Bq\ s}\right]$
1	3603	1334	4.64E−05	6.96E−05	5.16E−05
2	3466	1374	6.67E−05	1.05E−04	6.39E−05
3	1877	721	7.05E−05	1.22E−04	7.79E−05
4	1853	722	7.27E−05	5.41E−05	7.66E−05
5	3932	1004	5.10E−05	8.57E−05	4.79E−05
6	4228	1096	5.30E−05	1.32E−04	4.60E−05
7	4149	1155	6.46E−05	1.05E−04	5.63E−05
8	3495	915	5.78E−05	8.12E−05	5.70E−05
9	5309	1231	4.27E−05	8.71E−05	4.20E−05
Average	3546 ± 1104^b^	1061 ± 241^b^	5.84E−05 ± 1.08E−05^b^	9.35E−05 ± 2.48E−05^b^	5.77E−05 ± 1.28E−05^b^

^a^As reported in Fig. 2, ^b^ one standard deviation. ^c^ *V*_C_ = cellular volume, ^d^ *V*_N_ = nuclear volumes

**Fig. 5:**
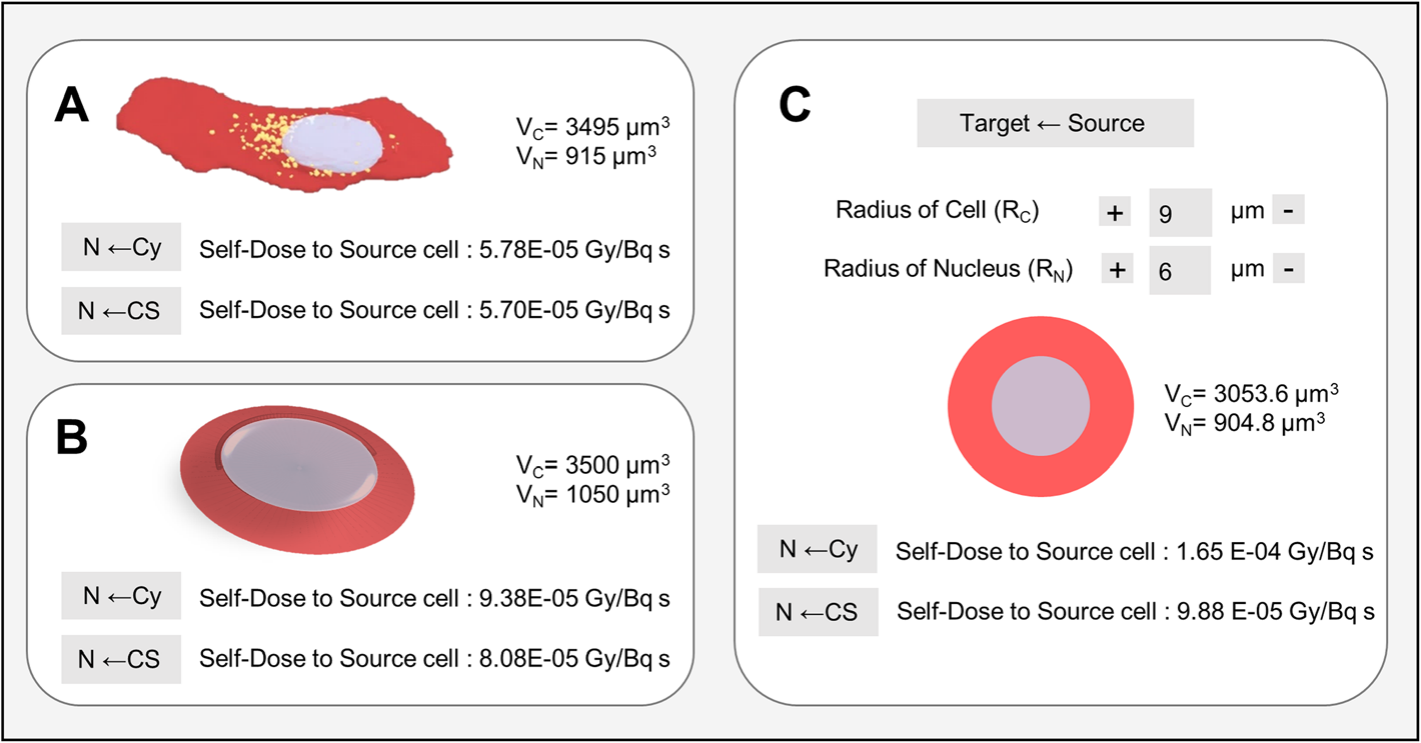
Effect of accurate shape modeling on the self-dose to the nucleus. **a** Example of a typical cell morphology with the corresponding *S* values from cell surface and cytoplasm. The same *S* values corresponding to the sphere **b** and truncated cone **c** when selecting similar volumes are reported for comparison. V_C_ and V_N_ indicate the cellular and nuclear volume, respectively

An example is shown comparing a typical cell morphology (Fig. 5a) with a truncated cone (Fig. 5b) and sphere (Fig. 5c) of similar volumes (*R*_c_ = 9μm; *R*_n_ = 6μm). Assuming the PM geometry as reference, the sphere morphology leads to an overestimation of the absorbed dose to the nucleus from cytoplasm and cell membrane of 65% and 42%, respectively; refining the cellular representation with a CSG shape, such as the truncated cone, decrease this discrepancy to 43% and 18%, respectively.

### Cell size and source location affects self *S* value

In previous studies, cytoplasm and membrane placements were compared for the spherical model. We have furthered explored this comparison using the more realistic truncated cone geometry, including the Golgi and combining the effect of cell dimension.

**Fig. 6:**
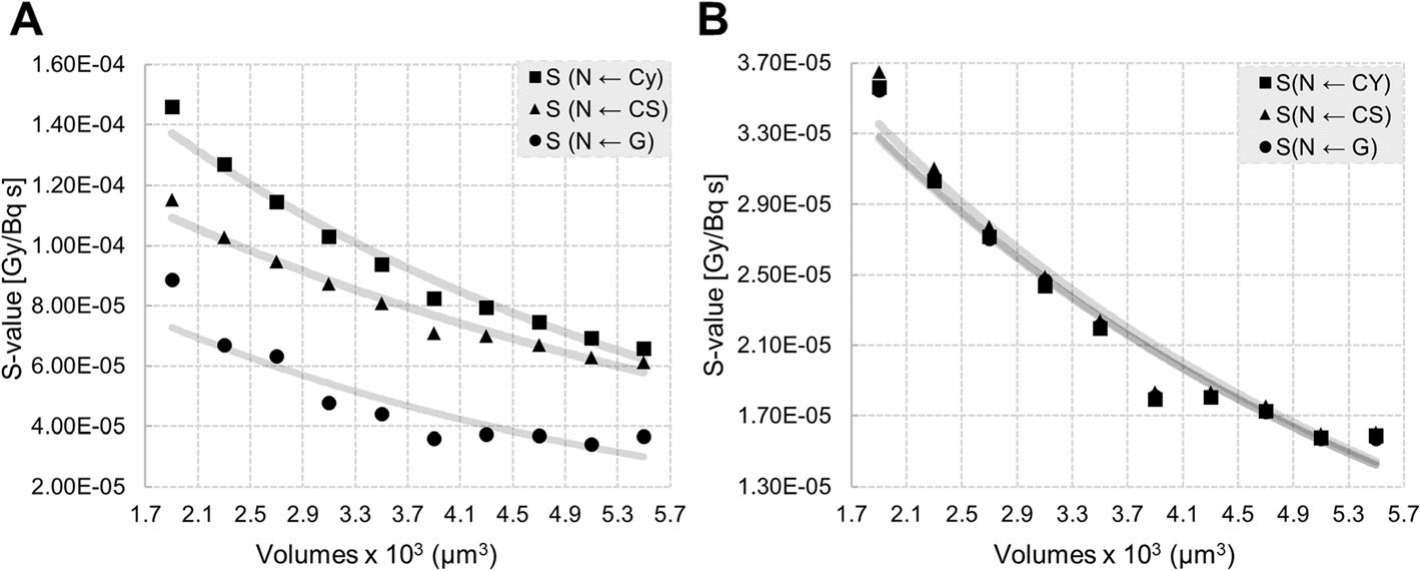
Effect of cellular volume and source location on the self-dose **a** and cross-dose **b** dose to nucleus. Square, triangle and circle correspond to the radioactive source distributed in cytoplasm, membrane and Golgi, respectively

Both *S*_N ← Cy_ and *S*_N ← CS_ decrease exponentially (*R*^2^ = 0.98 and *R*^2^ = 0.97, respectively) with increasing cell volumes. For instance, reducing the average cell volume (3500 μm^3^) to the smallest volume observed (1900 μm^3^), leads to an increase in the absorbed dose of 56% and 43% for cytoplasm and cell membrane, respectively. Whereas increasing its volume to the largest volume observed (5500 μm^3^) leads to a reduction in the self-dose of 30% for Cy and 24% for cell membrane (Fig. 6a; Additional file 1: Table S2).

Furthermore, the sub-cellular distribution of the radionuclide significantly affects the self-absorbed dose (*p* < 0.05 comparing cytoplasm and cell membrane). The nucleus receives a higher dose from the cytoplasm rather than from the cell membrane; specifically, the smaller the cell the larger the dose contribution given by the cytoplasm compared to the cell membrane, assuming the same activity in both compartments.

### Only cell size affects the cross *S* value

The same analysis reported above has been performed on the total cross-dose imparted by touching cells, as described in the “Parameter analysis of *S* values” section. As the volume increases the total cross-dose decreases exponentially. For instance, it increases on average with 63% when reducing the average cell volume to 1900 μm^3^ and reduces its value of 28% when increasing the average cell volume to 5500 μm^3^, showing no dependency on the source location (Fig. 6b; Additional file 1: Table S3).

The ratio of total cross- to self-dose is on average 23% ± 1% from cytoplasm to nucleus and 28% ± 2% from cell membrane to nucleus, depending on the cell dimension. Therefore, in a realistic flattened cell representation, such as the truncated cone, the self-dose outweighs the cross-dose.

### Golgi is modeled with polygonal mesh structures

After analyzing the impact of source location in the truncated cone geometry, we have compared these findings against the PM data reported above in order to assess when the implementation of voxelized/polygonal mesh structures is deemed necessary.

In the truncated cone representation, simplifying the Golgi as half-circular torus significantly underestimates the radiation potentially imparted by this organelle (Table 3). Indeed, the complex structure of the Golgi, made of several vesicles distributed at variable distances from the nucleus, makes an accurate CSG representation challenging. Therefore, only the PMs allowed to take into account the alleged translocation of lutetium-177 to the Golgi (*S*_(N ← G)_) accurately, resulting in an increased absorbed dose to the nucleus of + 64% (averaged over 9 PM models) with a maximum + 149% (corresponding to cell 6) when compared to a homogeneous distribution of the activity in the cytoplasm (*S*_(N ← CY)_) (Table 2).

**Table 3:** Comparison between the doses delivered by Golgi to nucleus for 2 geometrical assumptions: PM models and simplified CSG. The data are listed in increasing order of Golgi volume (*V*_g_)

PM *V*_g_ (μm^3^)	S_(N ← G)_ $\left[\frac{\mathrm{Gy}}{\mathrm{Bq}\ \mathrm{s}}\right]$	CSG *V*_g_ (μm^3^)	S_(N ← G)_ $\left[\frac{\mathrm{Gy}}{\mathrm{Bq}\ \mathrm{s}}\right]$
123	1.05E−04	110	3.67E−05
80	8.71E−05	94	3.70E−05
68	1.05E−04	86	3.75E−05
63	1.32E−04	78	3.60E−05
60	8.57E−05	70	4.40E−05
54	8.12E−05	62	4.78E−05
31	6.96E−05	54	6.32E−05
25	5.41E−05	46	6.71E−05
24	1.22E−04	38	8.87E−05
Average	(9.4±2.5) E−05	Average	(5.1±1.8) E−05

Cell volumes: 1900 ÷ 5500 μm^3^ and nucleus volumes: 570 ÷ 1652 μm^3^, corresponding to reference MIRD cell with *R*c = 8 ÷ 11 μm and *R*n = 6 ÷ 7 μm

### Cross-dose is negligible above 5 cells distance for equally spaced cells

In order to analyze the cross-dose imparted by non-neighboring cells and to model the multicellular monolayer geometry during the first 4 h exposure, we have investigated the effect of cell proximity. For this purpose, we compared the total cross-dose, when average-sized cells are plated at a given distance between each other with respect to the reference case of touching cells, using the truncated cone geometry. The total cross-dose, which is already a minor contribution of the total dose compared to the self-dose, is reduced with 82% at 1 cell diameter (average cell distance during the uptake experiments). At 5 cell diameters it becomes negligible (− 99% of reference value), leading to the assumption that the absorbed dose imparted from cells located further away from each other can be disregarded. Coherent with the previous findings, there is no significant difference in the total cross-dose between source locations (Additional file 1: Table S4).

### Nucleus placement does not affect *S* value for cytoplasm and cell membrane

In previous studies, the effect of non-concentric cell and nucleus morphology for the spherical cell model was reported. Therefore, we have explored the effect of nucleus placement in our truncated cone geometry.

Shifting the nucleus position has little influence (up to 4%) on the self- and cross-dose imparted by the cytoplasm or cell membrane to the nucleus. Instead, a dose reduction of 15–36% is observed when placing the source in the Golgi and shifting the nucleus away of 1–3 μm from it longitudinally (on *y*-axis), implying that modeling the Golgi few μm away from the nucleus could significantly affects the final absorbed dose calculation (Additional file 1: Table S5-S6).

## Dosimetry of clonogenic survival assay

### High-resolution time-activity cellular uptake curves

**Fig. 7:**
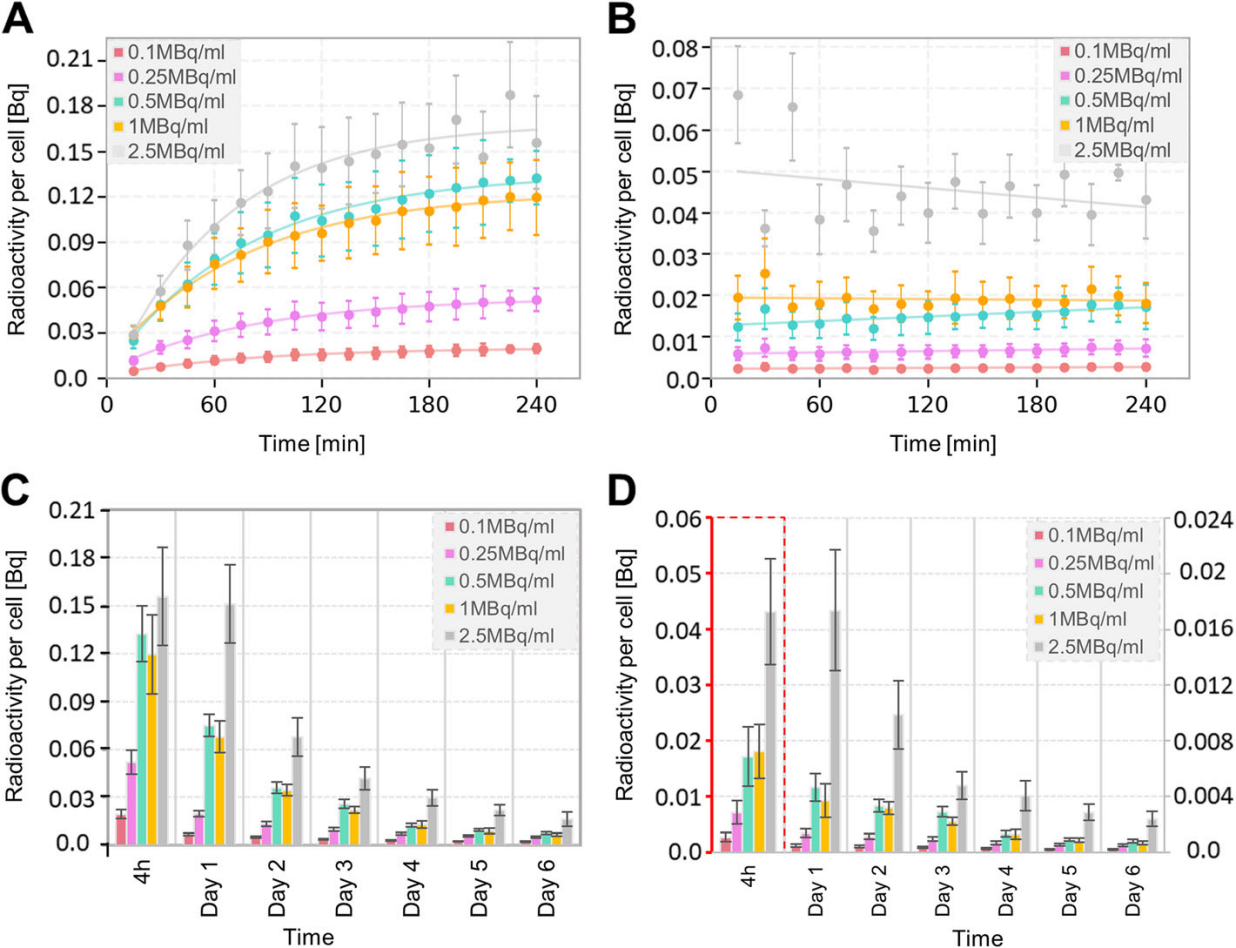
Activity quantification. Internalized **a** and **b** membrane bound activity curves per cell during the 4 h uptake. Internalized **c** and **d** membrane bound activity per cell at the end of the 4 h uptake and in each following day. The curves corresponding to 0.1 MBq/ml and 0.25 MBq/ml are extrapolated. The error bars indicate the SD of uptake data

The membrane-bound activity per cell saturates within the first 15 min thus showing a linear function with incubation time, whilst the internalized fraction reaches a plateau after 2 h (exponential association function; *R*^2^ > 0.95) (Fig. 7a, b). In addition, the decay-corrected membrane-bound and internalized activities per cell quantified each subsequent day show an exponentially decreasing trend (*R*^2^ > 0.94) (Fig. 7c, d; Additional file 1: Table S7).

After the first 4 h of incubation, lutetium-177 is released into the medium because of active or passive excretion and cell death (Additional file 1: Figure S1). The effective decay rate is 0.024 ± 0.002 h^−1^.

### MIRDcell predicts higher absorbed doses than PM models

**Fig. 8:**
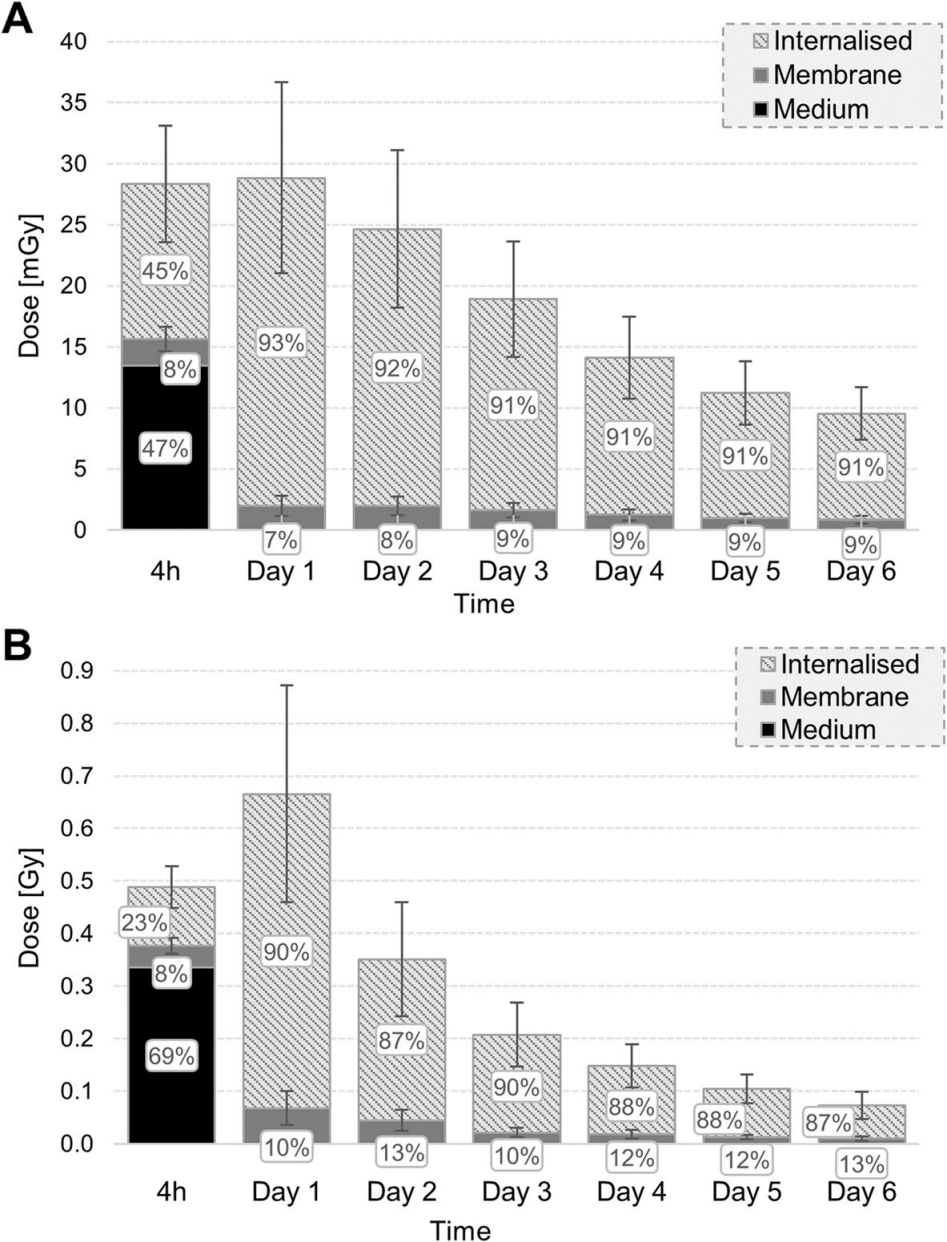
Absorbed dose contribution to the nucleus from unbound, membrane bound and internalized fraction of activity (in cytoplasm) through the experiment. **a** corresponds to 0.1 MBq/ml and **b** to 2.5 MBq/ml. Calculations were performed using the avarage *S* values of the 9 PM geometries, including the varying cross dose. The error bars indicate the SD of the absorbed dose

Following, the absorbed dose delivered by medium, cytoplasm/Golgi and cell membrane is calculated each day (Fig. 8) using the *S* values reported in Table 4 (see Additional file 1: Table S8 for the other geometrical assumptions). The medium fraction only contributes significantly (10–17%) to the dose imparted to the nucleus during the first 4 h (during incubation of the cells with ^177^Lu-DOTATATE). Furthermore, the higher the activity the more the influence of the unbound fraction on the absorbed dose during the first 4 h. Throughout the 6 days, the internalized fraction forms the main contribution to the absorbed dose to the nucleus because of the greater radionuclide uptake; its contribution corresponds to 82% and 73% of the total absorbed dose to the nucleus for the lowest and highest added activity, respectively. Eventually, the absorbed dose imparted by each cell compartment decreases, because of excretion, cell death, and physical decay of lutetium-177. The cumulative absorbed dose to the nucleus is reported for four scenarios: spheres, truncated cones and the PM geometry considering the internalized fraction either in the cytoplasm or Golgi (Table 5). Calculating the absorbed dose using each of the 9 PM models separately, we estimated that the final absorbed dose to the nucleus might differ up to ± 21% and ± 31% when the source is localized in cytoplasm or Golgi, respectively.

**Table 4:** *S* values calculated for the 4-h uptake (cellular distance of 1 diameter) and the 6-day colony forming (from single cells to clusters of increasing size) for polygonal mesh models and localization of the internalized source in cytoplasm or Golgi

	Polygonal mesh			Medium
Source	S_(N ← CS)_^a b^	S_(N ← Cy)_^a b^	S_(N ← G)_^a b^	
4-h uptake	6.17E−05	6.23E−05	9.75E−05	9.43E−12
Colony day-1	5.77E−05	5.84E−05	9.35E−05	9.43E−12
Colony day-2	6.07E−05	6.13E−05	9.65E−05	
Colony day-3	6.66E−05	6.70E−05	1.02E−04	
Colony day-4	7.30E−05	7.33E−05	1.08E−04	
Colony day-5	7.73E−05	7.76E−05	1.13E−04	
Colony day-6	8.01E−05	8.03E−05	1.15E−04	

^a^Mean monolayer *S* values (N ← CS, N ← Cy, N ← G), which is the sum of self and cross-dose to the nucleus in [Gy/(Bq s)]. ^b^*S*_(N ← CS)_±1.28E-05, *S*_(N ← Cy)_±1.08E-05 and *S*_(N ← G)_±2.48E−05. The SD is given only by the variation in the self-*S* value contribution

**Table 5:** Absorbed dose to the nucleus corresponding to each added activity for the 4 geometrical modeling assumptions

Activity [MBq/ml]	Sphere [*Gy*]	TC^a^ (Cy)^b^ [*Gy*]	PM (Cy)^b^ [*Gy*]	PM (G)^b^ [*Gy*]
0.1	0.42 ± 0.05	0.20 ± 0.02	0.14 ± 0.04	0.20 ± 0.07
0.25	1.08 ± 0.13	0.52 ± 0.06	0.35 ± 0.09	0.51 ± 0.18
0.5	2.77 ± 0.30	1.39 ± 0.15	0.92 ± 0.25	1.36 ± 0.47
1	2.63 ± 0.36	1.36 ± 0.18	0.92 ± 0.25	1.33 ± 0.47
2.5	5.64 ± 0.96	2.95 ± 0.47	2.04 ± 0.60	2.86 ± 1.08

^a^ *TC*=Truncated cone. ^b^ The localization of the internalized source is indicated in parenthesis

### ^177^Lu-DOTATATE and x-ray exposure cause different dose-responses

**Fig. 9:**
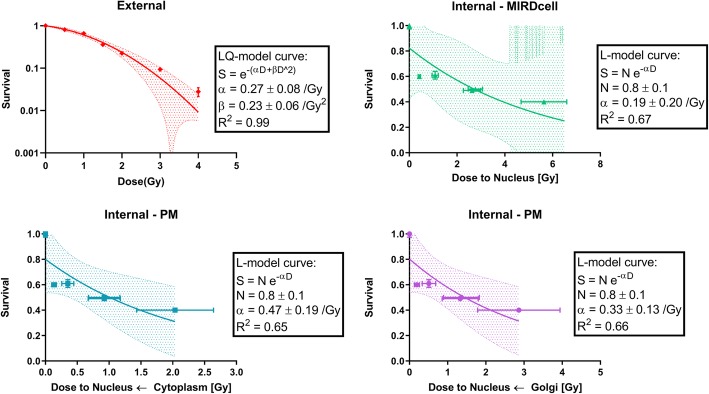
Dose-response curves corresponding to x-ray irradiation (top left) and 177Lu-DOTATATE (top right: MIRDcell, bottom left: PM with internalized source in cytoplasm, bottom right: PM with internalized source in Golgi). Each data point is plotted as the mean survival±SEM

After calculating the absorbed dose to the nucleus, we sought correlations with the experimental survival fractions (SF). The SF versus the absorbed dose to the nucleus after 86-keV x-ray irradiation and ^177^Lu-DOTATATE exposure (MIRDcell and PM models) is plotted in Fig. 9. The survival data of the x-ray irradiation could be fitted by the LQ-model (*R*^2^ = 0.99) with the following parameters α = 0.27 ± 0.08/Gy and *β* = 0.23 ± 0.06 /Gy^2^, *α*/*β* = 1.19 ± 0.67 Gy. Instead, for the ^177^Lu-DOTATATE survival data, the Akaike test showed as preferred fitting model the linear dose-response (*α*/*β* > 100 Gy); the corresponding *α* value for all the modeling assumptions are reported in Table 6. The absorbed doses calculated by implementing MIRDcell *S* values exceeded the dose-range of the x-ray irradiation.

**Table 6:** The LQ-model parameter *α* calculated for the x-ray irradiation and the 4 modeling assumptions adopted for the ^177^Lu-DOTATATE exposure (with *α*/*β* > 100 Gy): MIRDcell, truncated cone, and PM assuming the internalized source either in cytoplasm (PM-Cy) or Golgi (PM-G)

	x-ray	MIRDcell	Truncated cone	PM-Cy	PM-G
*α*$\left[\frac{1}{Gy}\right]$	0.27 ± 0.08	0.19 ± 0.20	0.32 ± 0.38	0.47 ± 0.19	0.33 ± 0.13
*R*^2^	0.99	0.67	0.66	0.65	0.66

## Discussion

For the evaluation of novel radiopharmaceuticals, an accurate assessment of the cell-absorbed dose is crucial to get more insight into the aspects enabling a clinically effective treatment. Therefore, we first systematically analyzed the impact of several cellular parameters on the self- and cross-dose contributions from intracellular radiation exposures. Next we developed a dosimetric model for the calculation of the average absorbed dose during 2D in vitro experiments including morphological (cell size, shape) and cell culture characteristics (proliferation, proximity variation, cell death) combined with detailed activity uptake kinetics.

For this purpose, we compared *S* values obtained for the same cellular shape in MCNP6.1 and Geant4-10.03 for concentric spheres (including MIRDcell) and truncated cones. The relative deviation between the codes was small (< 5%) and could be attributed to several factors: (1) the inclusion of δ-rays and energy-loss straggling in Monte Carlo calculations ignored by MIRDcell [10], (2) the use of a different cellular composition rather than water [28], (3) the use of different cross-sections and (4) *β*-spectra.

Receptor-mediated endocytosis of the radiolabeled peptides plays a crucial role in causing cytotoxic effects for *β*-emitters, because of the large number of *β* traversals (10000–20000) required for cell killing [29]. Therefore, the quantification of cellular uptake is a critical step to accurately estimate the effect of PRRT. However, the common protocol found in literature for determining the cellular uptake of radioactivity foresees one time point after saturation of the receptors [30], while our cellular uptake dataset allows for a more accurate quantification of the time integrated activity.

Many research groups have investigated the cellular uptake of radioactivity in cell populations and found lognormal distributions of activity, including a fraction of non-radiolabeled cells, which could lead to an increase in survival fractions. However, due to the lack of evidence on uptake heterogeneity in our cells, we assumed a uniform activity distribution among cells. Marcatili et al. investigated the impact of heterogeneity of uptake between cells and change in geometrical configurations for lutetium-177-labeled antibodies finding a variation of 24% on the cross-dose when randomizing both these parameters. When applying such an uncertainty on our data this would not significantly affect (< 5%) the absorbed dose, since in our study the cross-dose was found to be a minor contributor to the total absorbed dose. Instead, the heterogeneity of cells morphology and sub-cellular uptake resulted to be the major factors affecting the absorbed dose. Noticeably, if the fraction of labeled cells decreases, it becomes even more crucial to improve the cellular modeling to reproduce the subcellular distribution of the radionuclide and accurately estimate the self-dose [31].

Cellular modeling can significantly influence the proximity of the cell membrane and cytoplasm to the nucleus and thus the self *S* value [13]. Differences in *S* values between spherical and truncated cone geometry were found to reach 40%. However, accurately modeling cellular shapes by constructive solid geometries can represent a challenge for peculiar shapes. Hence, simplified representations, such as the truncated cone, should be used with caution in these cases. Using polygonal mesh structures we could model the cell membrane closer to the nucleus than in any other simplified constructive solid geometry representation, revealing that the absorbed dose delivered by the cell membrane is comparable to the cytoplasm for the same cumulated activity, conversely to spheres or truncated cone geometry results.

Furthermore, geometrical approximations of organelles such as the Golgi, where the SST_2_ receptor and hence possible the lutetium-177, is located upon internalization within 15 min, largely affect the absorbed dose to the nucleus as well. Modeling the Golgi with half circular torus close to the nucleus leads to a significant underestimation (up to 73%) of the absorbed dose compared to polygonal meshes. Remarkably, its structural arrangement and proximity to the nucleus increases the absorbed dose to the nuclear target compared to uniform radionuclide distribution in the cytoplasm. Noticeably, the characterization of the internalized source distribution and the geometry of the targeted cells would become even more critical when using alpha- or Auger-radiations [14], due to their short range.

The outcome of our parameter analysis confirmed that both cell size and source location affect the self *S* value, whilst the cross *S* value is affected only by the cell size in all the tested geometries [7, 31].

Besides a better characterization of the cellular morphology, Marcatili et al. [30] proved the necessity of modeling the whole cell culture geometry, such as cell proximity and colony formation when involving *β*-emitters, since changes in these parameters can significantly modify survival curves. We found that the cross-dose contribution to the total absorbed dose would be overestimated (+ 15%) if neighboring cells are modeled as is generally considered in past studies [8], rather than cells placed at 1 diameter as was done in our study. Moreover, during the follow-up days, we included the cross-dose contribution by the cellular clusters newly formed due to proliferation, which was not taken into consideration in past studies as well [29, 30]. We found out that the cross-dose contribution, within a cluster, increased linearly with the number of days, reaching 16% of the total absorbed dose. On the other hand, the neighboring clusters were located further away than the average range of lutetium-177 electrons from the cluster to which the target cell belongs, thus their cross-dose contribution could be neglected.

As expected, the absorbed dose to the nucleus seemed to better match the survival trend rather than the added activity; increasing the added activity from 0.5MBq/ml to 1MBq/ml did not alter the survival (~ 50%), because of the equivalent cumulated activities in the different cell compartments, which lead to similar absorbed doses to the nucleus. The absorbed dose to the nucleus was found to be 3-fold higher for spheres (MIRDcell) compared to the polygonal mesh structures.

Establishing the correlation between absorbed dose to the nucleus and clonogenic survival, we found out that the linear quadratic-model fits both ^177^Lu-DOTATATE (with *β* = 0) and x-ray irradiation survival data (with *β* ≠ 0). The linear-model relies on the assumption of a high *α*/*β* or a short sub-lethal damage repair half-life, thus ignoring the quadratic term of the linear quadratic-model. The heterogeneous dose delivery, the very low dose-rate (< 0.1 Gy/h) and the protracted exposure might account for the differences in the survival curves. Indeed, the low dose-rate could cause defects in the detection of low levels of DNA damage and the synchronization of cells in a radiosensitive cell cycle phase [1]. This could explain the significant drop of the survival in the low dose-range and thus, the alpha value discrepancy between x-ray and ^177^Lu-DOTATATE exposures. We neglected morphological or radiation sensitivity changes during proliferation (S-phase) as well as the effect of dose-rate variation between 6 days (150 to 7 mGy/h for 2.5 MBq/ml), which can play a significant role in PRRT [1]. In addition, further modeling refinement can be achieved by implementing more realistic biochemical mechanisms (advanced knowledge on source distribution within the cell compartments, pinocytosis, etc.) and studying absorbed doses on different cellular targets (e.g., cytoplasm [32], mitochondria [33], cell membrane [34], entire cell [35], and the Golgi apparatus [36]). Besides, we did not include repopulation, bystander effects, and changes in radio-sensitivity as well as in geometry and Golgi-placement [37] throughout the cell cycle. Although this was beyond the scope of the current study, the development of our cellular dosimetry model will enable to investigate these aspects.

At present, clinical dosimetry does not take the typical complexity and heterogeneity at the cellular or multi-cellular level into account. Instead, characterizing the absorbed dose on micro-scale, not only relying on averaged large-scale dosimetry, is necessary to assess the biological response and be able to implement this information on a clinical scale. The natural extension of these in vitro experiments could be the investigation of the ability of the linear dose-response model to predict tumor control probability in pre-clinical animal models of this particular cancer type.

## Conclusion

Altogether these results indicate that accurate modeling of cellular shapes and organelles in order to sample the radionuclide distribution is crucial to better estimate the absorbed dose to nucleus. Our dosimetry model mimics the experimental design of in vitro treatments, comprehending realistic cellular features, dynamic changes in proliferation, proximity variations, and cell death. Moreover, it suggests that ^177^Lu-DOTATATE treatment might be more effective than indicated by average spherical cell dosimetry.

## Supplementary information

**Additional file 1: Figure S1.** Unbound radioactivity in the medium for different radioactivity concentrations (0.1-2.5MBq/ml) during the 6 days follow-up. **Table S1.** Self S-values comparison between different morphological assumptions for the cell geometry. The shaded areas guide the comparison of subcellular S-value for similar volumes. **Table S2.** Effect of cellular volume and source location on the self-dose to the nucleus. **Table S3.** Effect of cellular volume on the total cross-dose to the nucleus from different source locations. **Table S4.** Effect of distance between cells on the total cross-dose for different source locations. **Table S5.** Effect of nucleus placement on the self-dose to nucleus for different source locations. **Table S6.** Effect of nucleus placement on the total cross-dose to nucleus. **Table S7.** Unbound (medium), membrane bound and internalized fractions of activity (per cell) for different radioactivity concentrations (0.1-2.5MBq/ml) during the first 4 h uptake and the next 6 days follow up. **Table S8.** S-values calculated for the 4 h uptake (cellular distance of 1 diameter) and the follo6 days colony forming (from single cells to clusters of increasing size) for different assumptions related to cellular geometry (truncated cone, PM and sphere) and localization of the internalized source (cytoplasm or Golgi for PM).

## Data Availability

The datasets supporting the conclusions of this article are included within the article and its additional file.

## Abbreviations

AIC: Akaike information criterion
CS: Cell surface
CSDA: Continuous-slowing-down approximation
CSG: Constructive solid geometry
Cy: Cytoplasm
EBRT: External beam radiotherapy
G: Golgi
LQ: Linear-quadratic
M: Medium
MIRD: Medical internal radiation dose
N: Nucleus
NET: Neuroendocrine tumors
PM: Polygonal mesh
PRRT: Peptide receptor radionuclide therapy
RPD: Relative percentage difference
RT: Room temperature
SD: Standard deviation
SF: Survival fraction
SST_2_: Somatostatin receptor type 2
TRT: Targeted radionuclide therapy
*V*_C_: Volume cell
*V*_Cy_: volume cytoplasm
*V*_G_: volume Golgi
*V*_N_: Volume nucleus
