# Supplementary material for: Cellular dosimetry of [177Lu]Lu-DOTA-[Tyr3]octreotate radionuclide therapy: the impact of modeling assumptions on the correlation with in vitro cytotoxicity
Source: EJNMMI Phys. 2020 Feb 10;7:8. doi: 10.1186/s40658-020-0276-5 (PMC7010903; doi:10.1186/s40658-020-0276-5)
Supplement: Supplementary file 1 — Additional file 1: Figure S1. Unbound radioactivity in the medium for different radioactivity concentrations (0.1-2.5MBq/ml) during the 6 days follow-up. Table S1. Self S-values comparison between different morphological assumptions for the cell geometry. The shaded areas guide the comparison of subcellular S-value for similar volumes. Table S2. Effect of cellular volume and source location on the self-dose to the nucleus. Table S3. Effect of cellular volume on the total cross-dose to the nucleus from different source locations. Table S4. Effect of distance between cells on the total cross-dose for different source locations. Table S5. Effect of nucleus placement on the self-dose to nucleus for different source locations. Table S6. Effect of nucleus placement on the total cross-dose to nucleus. Table S7. Unbound (medium), membrane bound and internalized fractions of activity (per cell) for different radioactivity concentrations (0.1-2.5MBq/ml) during the first 4 h uptake and the next 6 days follow up. Table S8. S-values calculated for the 4 h uptake (cellular distance of 1 diameter) and the follo6 days colony forming (from single cells to clusters of increasing size) for different assumptions related to cellular geometry (truncated cone, PM and sphere) and localization of the internalized source (cytoplasm or Golgi for PM). [file 40658_2020_276_MOESM1_ESM.docx]

**Additional FIGURES AND TABLES**


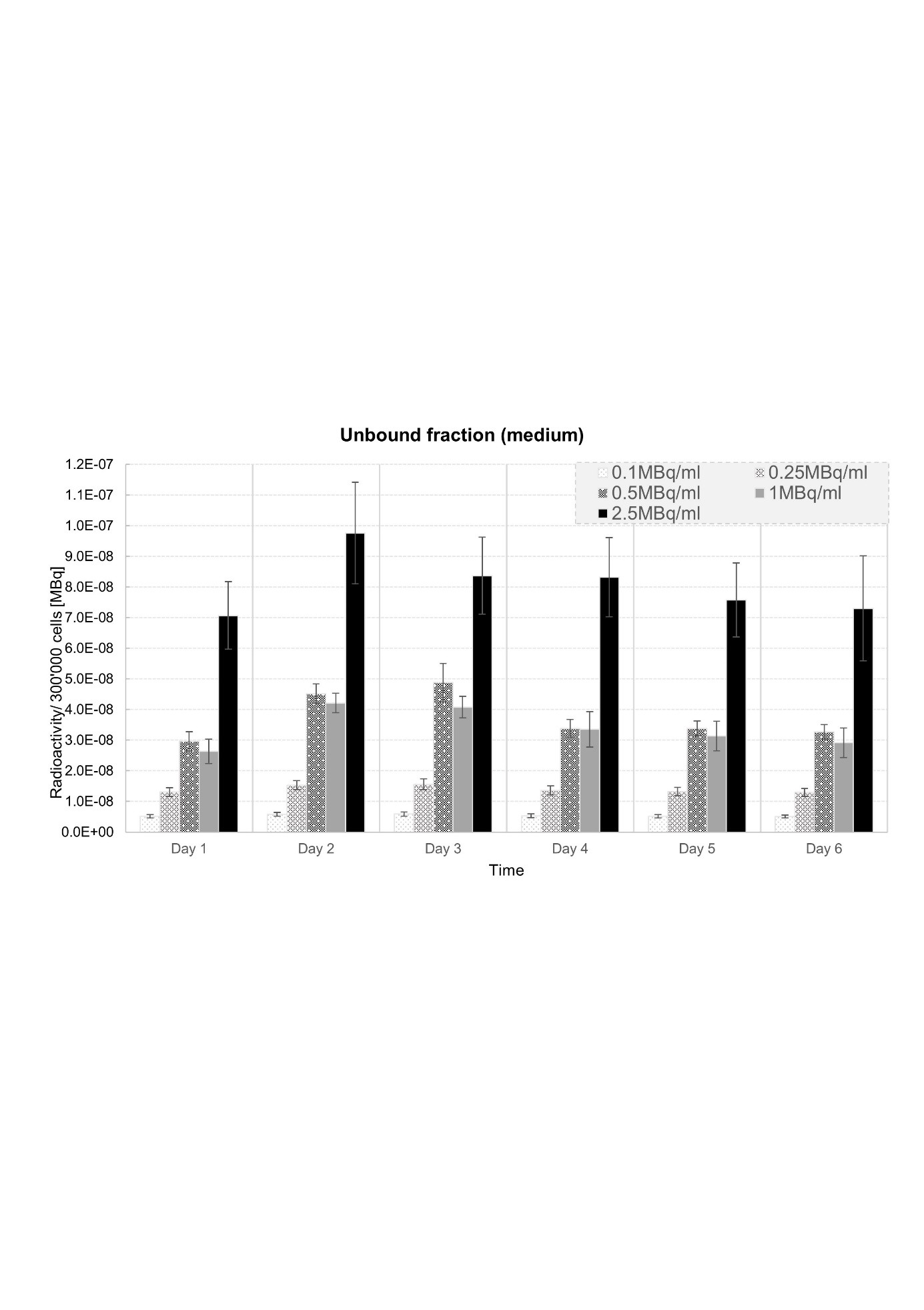


**Supplemental figure 1** Unbound radioactivity in the medium for different radioactivity concentrations (0.1-2.5MBq/ml) during the 6 days follow-up.

**Supplemental table 1** Self S-values comparison between different morphological assumptions for the cell geometry. The shaded areas guide the comparison of subcellular S-value for similar volumes.

| **Cell ID** | **Real cell** | | **MIRDcell^§^** | | | | **Truncated cone** | | | | **Voxelized** | | **Mesh structures** | | | |
| --- | --- | --- | --- | --- | --- | --- | --- | --- | --- | --- | --- | --- | --- | --- | --- | --- |
|  | **V_c_^*^** | **V_N_**^†^ | **Volumes / %ΔV**^‡^ | | **S_(N←Cy)_** | **S_(N←CS)_** | **Volumes /** **%ΔV**^‡^ | | **S_(N←Cy)_** | **S_(N←CS)_** | **Res. N^ǁ^** | **S_(N←Cy)_** | **Res.N^ǁ^** | **Res.G^¶^** | **S_(N←Cy)_** | **S_(N←CS)_** |
| 9 | 5309 | 1231 | V_c_ = 5575 / 5% | V_N_ = 905 / -27% | 1.18E-04 | 6.18E-05 | V_c_ = 5500 / 4% | V_N_ = 1652 / 34% | 6.59E-05 | 6.12E-05 | 1% | 3.58E-05 | -0.2% | 8.4% | 4.27E-05 | 4.20E-05 |
|  |  |  | V_c_ = 5575 / 5% | V_N_ = 1437 / 17% | 1.09E-04 | 6.38E-05 | V_c_ = 5100 / -4% | V_N_ = 1530 / 24% | 6.94E-05 | 6.29E-05 |  |  |  |  |  |  |
|  |  |  |  |  |  |  | V_c_ = 4700 / -11% | V_N_ = 1410 / 15% | 7.45E-05 | 6.70E-05 |  |  |  |  |  |  |
| 6 | 4228 | 1096 | V_c_ = 4189 / -1% | V_N_ = 905 / -17% | 1.38E-04 | 7.07E-05 | V_c_ = 4300 / 2% | V_N_ = 1290 / 18% | 7.94E-05 | 7.01E-05 | -4% | 6.08E-05 | -0.4% | 7.8% | 5.30E-05 | 4.60E-05 |
| 7 | 4149 | 1155 | V_c_ = 4189 / 1% | V_N_ = 1437 / 24% | 1.29E-04 | 8.04E-05 | V_c_ = 3900 / -6% | V_N_ = 1170 / 1% | 8.26E-05 | 7.09E-05 | 20% | 5.47E-05 | -0.3% | 2.9% | 6.46E-05 | 5.63E-05 |
| 5 | 3932 | 1004 |  |  |  |  | V_c_ = 3900 / -1% | V_N_ = 1170 / 17% | 8.26E-05 | 7.09E-05 | 8% | 3.80E-05 | -0.3% | 5.0% | 5.10E-05 | 4.79E-05 |
| 1 | 3603 | 1334 |  |  |  |  | V_c_ = 3500 / -3% | V_N_ = 1050 / -21% | 9.38E-05 | 8.08E-05 | -7% | 3.83E-05 | -3.1% | 11.3% | 4.64E-05 | 5.16E-05 |
| 8 | 3495 | 915 | V_c_ = 3054 / -13% | V_N_ = 905 / -1% | 1.65E-04 | 9.88E-05 | V_c_ = 3500 / 0% | V_N_ = 1050 / 15% | 9.38E-05 | 8.08E-05 | 2% | 4.96E-05 | -3.4% | 4.5% | 5.78E-05 | 5.70E-05 |
| 2 | 3466 | 1374 | V_c_ = 3054 / -12% | V_N_ = 1437 / 5% | 1.55E-04 | 1.05E-04 | V_c_ = 3500 / 1% | V_N_ = 1050 / -24% | 9.38E-05 | 8.08E-05 | 4% | 5.28E-05 | 0.9% | 2.1% | 6.67E-05 | 6.39E-05 |
|  |  |  |  |  |  |  | V_c_ = 3100 / -11% | V_N_ = 930 / -32% | 1.03E-04 | 8.73E-05 |  |  |  |  |  |  |
|  |  |  |  |  |  |  | V_c_ = 2700 / -22% | V_N_ = 810 / -41% | 1.15E-04 | 9.47E-05 |  |  |  |  |  |  |
| 3 | 1877 | 721 | V_c_ = 2145 / 14% | V_N_ = 905 / 25% | 2.03E-04 | 1.32E-04 | V_c_ = 2300 / 23% | V_N_ = 690 / -4% | 1.27E-04 | 1.03E-04 | -21% | 5.98E-05 | -2.0% | 11.9% | 7.05E-05 | 7.79E-05 |
| 4 | 1853 | 722 |  |  |  |  | V_c_ = 2300 / 24% | V_N_ = 690 / -4% | 1.27E-04 | 1.03E-04 | -24% | 5.59E-05 | -1.3% | 10.3% | 7.27E-05 | 7.66E-05 |
|  |  |  |  |  |  |  | V_c_ = 1900 / 3% | V_N_ = 570 / -21% | 1.46E-04 | 1.15E-04 |  |  |  |  |  |  |
| **Average** | **3546** | **1061** |  |  | **1.45E-04** | **8.75E-05** |  |  | **9.67E-05** | **8.20E-05** |  |  |  |  | **5.84E-05** | **5.77E-05** |
| SD | 1104 | 241 |  |  | 3.21E-05 | 2.57E-05 |  |  | 2.41E-05 | 1.66E-05 |  |  |  |  | 1.08E-05 | 1.28E-05 |
| ^*^ Cell volume, ^†^ Nucleus volume, ^‡^ The relative volumetric percentage differences are evaluated against the ones given by microscopic images (ImageJ) and reported in the first column, ^§^ The nucleus radius is either 6 or 7 μm, whereas the cell radius ranges from 8 to 11 μm, in order to have values comparable to the real cellular volumes, **^ǁ^** The resolution parameter is evaluated as relative percentage difference of the nucleus to cell ratio compared to the ones given by the microscopic images, ^¶^ The resolution parameter is evaluated as relative percentage difference of the Golgi to cell ratio compared to the ones given by the microscopic images, ^#^ Volumes are expressed in μm^3^, ^**^ S-values in $\frac{\mathrm{Gy}}{Bq\times s}$. | | | | | | | | | | | | | | | | |

**Supplemental table 2** Effect of cellular volume and source location on the self-dose to the nucleus.

| Volume x 10^3^  (μm^3^) | S _(N ← Cy)_  $\frac{\boldsymbol{Gy}}{\boldsymbol{Bq\times s}}$ | S _(N ← CS)_  $\frac{\boldsymbol{Gy}}{\boldsymbol{Bq\times s}}$ | S _(N ← G)_  $\frac{\boldsymbol{Gy}}{\boldsymbol{Bq\times s}}$ |
| --- | --- | --- | --- |
| 5.5 | 6.59E-05 | 6.12E-05 | 3.67E-05 |
| 5.1 | 6.94E-05 | 6.29E-05 | 3.41E-05 |
| 4.7 | 7.45E-05 | 6.70E-05 | 3.70E-05 |
| 4.3 | 7.94E-05 | 7.01E-05 | 3.75E-05 |
| 3.9 | 8.26E-05 | 7.09E-05 | 3.60E-05 |
| 3.5 | 9.38E-05 | 8.08E-05 | 4.40E-05 |
| 3.1 | 1.03E-04 | 8.73E-05 | 4.78E-05 |
| 2.7 | 1.15E-04 | 9.47E-05 | 6.32E-05 |
| 2.3 | 1.27E-04 | 1.03E-04 | 6.71E-05 |
| 1.9 | 1.46E-04 | 1.15E-04 | 8.87E-05 |

**Supplemental table 3** Effect of cellular volume on the total cross-dose to the nucleus from different source locations.

| Vol x 10^3^  (μm^3^) | S_(N ← CY)_  $\frac{\boldsymbol{Gy}}{\boldsymbol{Bq\times s}}$ | S_(N ← CS)_  $\frac{\boldsymbol{Gy}}{\boldsymbol{Bq\times s}}$ | S_(N ← G)_  $\frac{\boldsymbol{Gy}}{\boldsymbol{Bq\times s}}$ |
| --- | --- | --- | --- |
| 5.5 | 1.59E-05 | 1.61E-05 | 1.57E-05 |
| 5.1 | 1.58E-05 | 1.60E-05 | 1.57E-05 |
| 4.7 | 1.73E-05 | 1.75E-05 | 1.72E-05 |
| 4.3 | 1.80E-05 | 1.83E-05 | 1.81E-05 |
| 3.9 | 1.79E-05 | 1.83E-05 | 1.81E-05 |
| 3.5 | 2.20E-05 | 2.24E-05 | 2.21E-05 |
| 3.1 | 2.44E-05 | 2.49E-05 | 2.47E-05 |
| 2.7 | 2.72E-05 | 2.77E-05 | 2.70E-05 |
| 2.3 | 3.04E-05 | 3.10E-05 | 3.04E-05 |
| 1.9 | 3.56E-05 | 3.65E-05 | 3.55E-05 |

**Supplemental table 4** Effect of distance between cells on the total cross-dose for different source locations.

| Distance | S_(N←Cy)_  $\frac{\boldsymbol{Gy}}{\boldsymbol{Bq\times s}}$ | S_(N←CS)_  $\frac{\boldsymbol{Gy}}{\boldsymbol{Bq\times s}}$ | S_(N←G)_  $\frac{\boldsymbol{Gy}}{\boldsymbol{Bq\times s}}$ | %Δ̅S̅_(DIST-REF)_^⸶^ |
| --- | --- | --- | --- | --- |
| Ref.^*^ | 2.20E-05 | 2.24E-05 | 2.21E-05 | 0% |
| 0.3r | 1.54E-05 | 1.56E-05 | 1.55E-05 | -30.13% |
| 0.5r | 1.25E-05 | 1.27E-05 | 1.26E-05 | -43.12% |
| 1r | 8.01E-06 | 8.11E-06 | 8.08E-06 | -63.62% |
| 1.5r | 5.45E-06 | 5.51E-06 | 5.45E-06 | -75.33% |
| 2r | 3.96E-06 | 3.99E-06 | 3.95E-06 | -82.10% |
| 2.5r | 2.98E-06 | 3.00E-06 | 2.97E-06 | -86.55% |
| 3r | 2.26E-06 | 2.27E-06 | 2.26E-06 | -89.78% |
| 4r | 1.42E-06 | 1.44E-06 | 1.43E-06 | -93.54% |
| 6r | 6.68E-07 | 6.76E-07 | 6.68E-07 | -96.97% |
| 10r | 2.15E-07 | 2.17E-07 | 2.16E-07 | -99.03% |
| ^*^ Reference case of touching cells, ^⸶^Average percentage difference between total cross-dose for touching cells (Ref.) and for equally spaced cells at each reported distance | | | | |

**Supplemental table 5** Effect of nucleus placement on the self-dose to nucleus for different source locations.

| Shift  (μm) | S_(N←Cy)_  $\frac{\boldsymbol{Gy}}{\boldsymbol{Bq\times s}}$ | S_(N←CS)_  $\frac{\boldsymbol{Gy}}{\boldsymbol{Bq\times s}}$ | S_(N←G)_  $\frac{\boldsymbol{Gy}}{\boldsymbol{Bq\times s}}$ |
| --- | --- | --- | --- |
| z=-0.15 | 9.38E-05 | 8.13E-05 | 4.44E-05 |
| z=-0.30 | 9.30E-05 | 8.22E-05 | 4.47E-05 |
| y=+1 | 9.34E-05 | 8.07E-05 | 3.75E-05 |
| y=+2 | 9.22E-05 | 8.04E-05 | 3.24E-05 |
| y=+3 | 9.01E-05 | 8.01E-05 | 2.82E-05 |
| Reference | 9.38E-05 | 8.08E-05 | 4.40E-05 |

**Supplemental table 6** Effect of nucleus placement on the total cross-dose to nucleus.

| Shift  (μm) | S_(N←Cy)_  $\frac{\boldsymbol{Gy}}{\boldsymbol{Bq\times s}}$ | S_(N←CS)_  $\frac{\boldsymbol{Gy}}{\boldsymbol{Bq\times s}}$ | S_(N←G)_  $\frac{\boldsymbol{Gy}}{\boldsymbol{Bq\times s}}$ |
| --- | --- | --- | --- |
| z=-0.15 | 2.20E-05 | 2.25E-05 | 2.21E-05 |
| z=-0.30 | 2.20E-05 | 2.25E-05 | 2.21E-05 |
| y=+1 | 2.20E-05 | 2.24E-05 | 2.23E-05 |
| y=+2 | 2.21E-05 | 2.25E-05 | 2.26E-05 |
| y=+3 | 2.22E-05 | 2.27E-05 | 2.29E-05 |
| Reference | 2.20E-05 | 2.24E-05 | 2.21E-05 |

|  | |  | |  | |  | |  | |  | |  | |  |  |
| --- | --- | --- | --- | --- | --- | --- | --- | --- | --- | --- | --- | --- | --- | --- | --- |
| **Activity** | | **4 h** | | **Day 1** | | **Day 2** | | **Day 3** | | **Day 4** | | **Day 5** | | **Day 6** |  |
|  | |  | |  | |  | |  | |  | |  | |  |  |
| **Medium** | | | | | | | | | | | | | | |  |
| **0.1MBq/ml** |  | | 5.17E-09±5.64E-10 | | 5.78E-09±5.83E-10 | | 5.87E-09±6.66E-10 | | 5.33E-09±5.80E-10 | | 5.16E-09±5.40E-10 | | 5.05E-09±5.26E-10 | | |
| **0.25MBq/ml** |  | | 1.30E-08±1.42E-09 | | 1.53E-08±1.46E-09 | | 1.56E-08±1.81E-09 | | 1.36E-08±1.45E-09 | | 1.32E-08±1.33E-09 | | 1.30E-08±1.29E-09 | | |
| **0.5MBq/ml** |  | | 2.96E-08±3.15E-09 | | 4.52E-08±3.20E-09 | | 4.88E-08±6.27E-09 | | 3.38E-08±2.93E-09 | | 3.38E-08±2.43E-09 | | 3.27E-08±2.33E-09 | | |
| **1MBq/ml** |  | | 2.64E-08±3.99E-09 | | 4.22E-08±3.15E-09 | | 4.08E-08±3.51E-09 | | 3.35E-08±5.75E-09 | | 3.14E-08±4.83E-09 | | 2.91E-08±4.79E-09 | | |
| **2.5MBq/ml** |  | | 7.07E-08±1.11E-08 | | 9.76E-08±1.66E-08 | | 8.37E-08±1.26E-08 | | 8.32E-08±1.29E-08 | | 7.58E-08±1.21E-08 | | 7.30E-08±1.71E-08 | | |
| **Membrane** | | | | | | | | | | | | | | |  |
| **0.1MBq/ml** | 2.74E-09±7.89E-10 | | 5.02E-10±1.04E-10 | | 4.34E-10±7.88E-11 | | 3.65E-10±6.85E-11 | | 2.82E-10±5.18E-11 | | 2.35E-10±4.22E-11 | | 2.16E-10±3.99E-11 | | |
| **0.25MBq/ml** | 7.15E-09±2.11E-09 | | 1.39E-09±2.90E-10 | | 1.15E-09±2.00E-10 | | 9.55E-10±1.79E-10 | | 6.92E-10±1.25E-10 | | 5.65E-10±9.81E-11 | | 5.14E-10±9.33E-11 | | |
| **0.5MBq/ml** | 1.71E-08±5.35E-09 | | 4.65E-09±9.86E-10 | | 3.34E-09±4.46E-10 | | 2.91E-09±3.57E-10 | | 1.34E-09±1.99E-10 | | 9.47E-10±1.11E-10 | | 8.14E-10±1.29E-10 | | |
| **1MBq/ml** | 1.81E-08±4.88E-09 | | 3.70E-09±1.21E-09 | | 3.18E-09±4.58E-10 | | 2.23E-09±2.74E-10 | | 1.29E-09±3.46E-10 | | 8.86E-10±1.54E-10 | | 6.86E-10±1.29E-10 | | |
| **2.5MBq/ml** | 4.31E-08±9.45E-09 | | 1.74E-08±4.31E-09 | | 9.85E-09±2.45E-09 | | 4.79E-09±9.85E-10 | | 4.05E-09±1.08E-09 | | 2.87E-09±5.89E-10 | | 2.40E-09±5.51E-10 | | |
| **Internalized** | | | | | | | | | | | | | | |  |
| **0.1MBq/ml** | 1.94E-08±2.93E-09 | | 6.79E-09±7.27E-10 | | 5.01E-09±5.49E-10 | | 3.87E-09±4.37E-10 | | 2.93E-09±3.34E-10 | | 2.44E-09±2.78E-10 | | 2.22E-09±2.59E-10 | | |
| **0.25MBq/ml** | 5.18E-08±7.61E-09 | | 1.96E-08±2.04E-09 | | 1.31E-08±1.41E-09 | | 9.92E-09±1.12E-09 | | 7.10E-09±8.01E-10 | | 5.84E-09±6.48E-10 | | 5.22E-09±5.99E-10 | | |
| **0.5MBq/ml** | 1.32E-07±1.76E-08 | | 7.50E-08±6.96E-09 | | 3.59E-08±3.51E-09 | | 2.57E-08±2.82E-09 | | 1.25E-08±1.20E-09 | | 9.59E-09±7.30E-10 | | 7.54E-09±7.22E-10 | | |
| **1MBq/ml** | 1.20E-07±2.48E-08 | | 6.78E-08±9.97E-09 | | 3.45E-08±3.52E-09 | | 2.20E-08±1.95E-09 | | 1.28E-08±2.13E-09 | | 8.93E-09±1.61E-09 | | 6.79E-09±9.47E-10 | | |
| **2.5MBq/ml** | 1.56E-07±3.06E-08 | | 1.51E-07±2.43E-08 | | 6.77E-08±1.21E-08 | | 4.18E-08±7.05E-09 | | 2.96E-08±5.11E-09 | | 2.19E-08±3.51E-09 | | 1.62E-08±4.63E-09 | | |

**Supplemental table 7** Unbound (medium), membrane bound and internalized fractions of activity (per cell) for different radioactivity concentrations (0.1-2.5MBq/ml) during the first 4 h uptake and the next 6 days follow up.

**Supplemental table 8** S-values calculated for the 4 h uptake (cellular distance of 1 diameter) and the follo6 days colony forming (from single cells to clusters of increasing size) for different assumptions related to cellular geometry (truncated cone, PM and sphere) and localization of the internalized source (cytoplasm or Golgi for PM).

|  | **Truncated cone** | | | | **Polygonal mesh** | | | | | | **Sphere** | | | **All** |
| --- | --- | --- | --- | --- | --- | --- | --- | --- | --- | --- | --- | --- | --- | --- |
| **Source** | **S_(N←CS)_^*^** | **S_(N←Cy)_^*^** | | **S_(N←CS)_^*^** ^†^ | | **S_(N←Cy)_^*^** ^†^ | | **S_(N←G)_^*^** ^†^ | | **S_(N←CS)_^*^** | | **S_(N←Cy)_^*^** | | **Medium** |
|  |  | | | | | | | | | | | | | |
| **4 h uptake** | 8.48E-05 | | 9.78E-05 | | 6.17E-05 | | 6.23E-05 | | 9.75E-05 | | 1.37E-04 | | 2.03E-04 | 9.43E-12 |
|  |  | |  | |  | |  | |  | |  | |  |  |
| **Colony Day-1** | 8.08E-05 | | 9.38E-05 | | 5.77E-05 | | 5.84E-05 | | 9.35E-05 | | 9.88E-05 | | 1.65E-04 | 9.43E-12 |
| **Colony Day-2** | 8.39E-05 | | 9.68E-05 | | 6.07E-05 | | 6.13E-05 | | 9.65E-05 | | 1.22E-04 | | 1.88E-04 |  |
| **Colony Day-3** | 8.97E-05 | | 1.02E-04 | | 6.66E-05 | | 6.70E-05 | | 1.02E-04 | | 1.70E-04 | | 2.33E-04 |  |
| **Colony Day-4** | 9.61E-05 | | 1.09E-04 | | 7.30E-05 | | 7.33E-05 | | 1.08E-04 | | 2.26E-04 | | 2.88E-04 |  |
| **Colony Day-5** | 1.00E-04 | | 1.13E-04 | | 7.73E-05 | | 7.76E-05 | | 1.13E-04 | | 2.68E-04 | | 3.30E-04 |  |
| **Colony Day-6** | 1.03E-04 | | 1.16E-04 | | 8.01E-05 | | 8.03E-05 | | 1.15E-04 | | 2.95E-04 | | 3.57E-04 |  |

^*^Mean monolayer S-values (N←CS, N←Cy, N←G), which is the sum of self and cross-dose to the nucleus in [Gy/(Bq s)]

^†^S_(N←CS)_±1.28E-05, S_(N←Cy)_±1.08E-05 and S_(N←G)_±2.48E-05
